# Height and body mass index in relation to cancer of the small intestine in two million Norwegian men and women

**DOI:** 10.1038/sj.bjc.6602789

**Published:** 2005-09-20

**Authors:** T Bjørge, S Tretli, A Engeland

**Affiliations:** 1 Department of Public Health and Primary Health Care, Section for Epidemiology and Medical Statistics, University of Bergen, Bergen N-5018, Norway; 2 The Cancer Registry of Norway, Institute of Population-Based Cancer Research, Montebello, Oslo N-0310, Norway; 3 Division of Epidemiology, Norwegian Institute of Public Health, Oslo N-0403, Norway

**Keywords:** body mass index, cancer of the small intestine, cohort study, Norway

## Abstract

The present study aimed at exploring the relations between body mass index (BMI, (weight in kilograms)/(height in meters)^2^) and stature and cancer of the small intestine (1162 cases) in a large Norwegian cohort (of two million) with measured height and weight. Elevated BMI in males and increasing height in both sexes were associated with a moderately increased risk of cancer of the small intestine.

Overweight or obesity has been linked to an increased risk of several site-specific cancers, including colorectal cancers (Engeland *et al*, 2005). A large cohort study of male US veterans found a significant excess risk of small intestine cancer, especially of the duodenum, among obese white veterans (Samanic *et al*, 2004). Also, a recent cohort study of obese individuals in Sweden reported a significantly elevated risk of cancer of the small intestine, but only among men (Wolk *et al*, 2001).

The aim of the present study was to explore the associations between body mass index (BMI) and height and the risk of cancers of the small intestine in a very large Norwegian cohort of both men and women with a long follow-up.

## STUDY POPULATION

The study population has been described in detail elsewhere, (Engeland *et al*, 2004).Briefly in a series of health surveys during 1963–2001, height and weight were measured in a standardised way by a trained staff in 2 001 727 persons (963 709 men and 1 038 018 women) aged 20–74 years. Deaths, emigrations and cases of cancer of the small intestine (International Classification of Diseases, seventh revision (ICD-7): 152) in this cohort were identified by linkage to the Death Registry at Statistics Norway (Statistics Norway, 2005) and to the Cancer Registry of Norway (The Cancer Registry of Norway, 2005). Both these registries are population based and are covering the entire Norwegian population. A unique 11-digit identification number assigned to all individuals living in Norway after 1960 simplified the linkages.

In the present study, only histologically verified cancers of the small intestine were included. Persons with a diagnosis of cancer of the small intestine, prior to the height and weight measurements, were excluded. Consequently, 32 persons were excluded in the analyses. In the analyses, the persons in the cohort were followed up from the date of measurement until the date of cancer diagnosis, emigration, age 100 years, death or until 31 December 2003. Altogether, 2 001 695 persons were eligible for the study. A small number of these (39 men and 32 women) were lost to follow-up.

## STATISTICAL METHODS

Cox's proportional-hazards regression models (Cox and Oakes, 1984), with time since measurement as the time variable, were fitted to obtain relative risk (RR) estimates of cancer. In the analyses, categorised variables for age at measurement, year of birth, BMI ((weight in kilograms)/(height in meters)^2^) and height were included. BMI was categorised using the WHO categorisation (World Health Organization Consultation on Obesity, 1998): BMI <18.5 (underweight), 18.5–24.9 (normal), 25.0–29.9 (preobese/overweight) and ⩾30.0 (obese).

Analyses were performed including BMI and height, respectively, as continuous variables to test for trend in the risk of cancer of the small intestine by BMI and height.

Separate analyses were performed for cancer at subsites of the small intestine and for different histologies. Lymphomas were not included in the total number of cases in the present study.

All these analyses were performed with the statistical program package SPSS (SPSS Inc., 2001). The results were presented as RR of cancer with 95% confidence intervals (CI).

## RESULTS

The 2 001 624 persons (963 655 men and 1 037 969 women) included in this study were followed for on average 23 years (range 0–41), constituting 47 million person-years (Table 1). The mean age at measurement was 44 years. The proportions of underweight were 0.7 and 2.0% in men and women, respectively, while the proportions of obese were 6 and 13%. During follow-up, 1162 cases of small intestine cancer were diagnosed among the study subjects. The mean age at diagnosis was 68 years, and the cases were measured on average 20 years prior to diagnosis. Of all small intestine cancer cases, 50% were carcinoids and 35% were adenocarcinomas.

An increase in small intestine cancer risk was seen with increasing BMI in men and with increasing height in both sexes (Table 2). The RR associated with a one-unit increase in BMI was 1.03 (95% CI: 1.01–1.06) in men. The RR associated with a 10 cm increase in height was 20% (95% CI: 5–37%) in men and 20% (95% CI: 4–39%) in women. A tendency towards an increase in risk with increasing BMI was seen for cancers of the duodenum in both sexes. Compared with men with normal BMI, overweight and obese men had an RR of carcinoids of 1.36 (95% CI: 1.07–1.74) and 1.63 (95% CI: 1.01–2.65), respectively. The risk of carcinoids increased with increasing height in both sexes. The RR associated with a 10 cm increase in height was 22% (95% CI: 1–47%) in men and 36% (95% CI: 11–68%) in women.

To exclude the possibility that at the time of BMI measurement weight was influenced by the presence of an undiagnosed small intestine cancer, we analysed the data after omitting the first 5 years of follow-up. Similar results were found.

## DISCUSSION

In the present study, a Norwegian cohort of more than two million men and women was followed prospectively with regard to the risk of small intestine cancer for an average of 23 years. More than 1150 cancer cases were observed, and the risk increased with BMI in men and with height in both sexes.

Our study subjects were recruited from population-based studies with high attendance, and the measurements were performed in a standardised way. Use of a unique identification number and population-based registries on deaths, emigrations and cancer incidence ascertained an almost complete follow-up of the study subjects (2 001 624 persons). Only 71 persons were lost to follow-up. By the end of follow-up, 60% of the persons were alive without a diagnosis of small intestine cancer, 40% were dead and 0.1% had small intestine cancer.

Large prospective studies have shown a significant association between obesity and several cancers (Bianchini *et al*, 2002; Calle *et al*, 2003). The International Agency for Research on Cancer has classified the evidence of a causal link as ‘sufficient’ for cancers of the colon, postmenopausal breast cancer, endometrial cancer, renal cell carcinoma and adenocarcinoma of the oesophagus (IARC Working Group on the Evaluation of Cancer-Preventive Strategies, 2002). We have previously, in the present data set, reported on a positive association between BMI and several site-specific cancers, including colorectal cancer (Engeland *et al*, 2005). In the present study, an increase in the risk of small intestine cancer was seen in men, and there was a tendency towards an increased risk of cancers of the duodenum in both sexes.

Very few studies have examined the association between body weight and less common cancers, such as cancer of the small intestine. A prospective study of obesity and cancer risk in Sweden showed a significant risk elevation for cancers of the small intestine compared with the expected number of cases (SIR=2.8, 95% CI=1.6–4.5). The excess risk was restricted to males (Wolk *et al*, 2001). The number of observed cases, however, was small (nine male and eight female cases). A cohort study of military veterans in the United States revealed a significant excess of small intestine cancer among obese compared with those not identified as obese (Samanic *et al*, 2004). The excess risk was only observed among white veterans, and was most pronounced for the duodenum.

A relatively large case–control study (430 cases) on risk factors for small intestine cancer found no association between BMI and cancer of the small intestine (Chow *et al*, 1993). Further, data from two Italian hospital-based case–control studies suggested an inverse relationship (Negri *et al*, 1999). However, only 23 cases were examined.

Cancer of the small intestine is rare, and relatively little is known about its aetiology. Risk factors for small bowel cancer, however, include dietary factors, cigarette smoking, alcohol intake and different medical conditions (Neugut *et al*, 1998). Further, adenocarcinomas of the small and large bowel share several common features, and it has been shown that there is a strong similarity between the dietary correlates of risk of adenocarcinoma of the small intestine and colon cancer (Negri *et al*, 1999).

BMI has been associated with an increased risk of colon cancer, most pronounced among men. It has been suggested that there is an effect of factors related to adiposity on the promotion of colon cancer, and a possible counteracting effect on these factors by estrogens (IARC Working Group on the Evaluation of Cancer-Preventive Strategies, 2002). Similar mechanisms might be involved in the aetiology of small intestine cancer, in particular adenocarcinomas of the small intestine.

In the present study, there was an increase in small intestine cancer risk with increasing height. Very few studies have had the possibility to examine the association between height and small intestine cancer risk. A large number of studies have, however, reported on a positive association between height and cancer risk at different other sites, for instance, colorectal cancer, prostate cancer and breast cancer (Gunnell *et al*, 2001). The associations reported have been relatively weak – the tallest individuals have been at a 20–60% increased risk compared with the shortest. The association has been explained by genes or by prenatal/childhood exposures. It has, however, also been demonstrated that raised levels of insulin-like growth factor 1 are associated with increased risk of different cancers (Gunnell *et al*, 2001).

In summary, in this large Norwegian cohort, the risk of cancer of the small intestine increased with BMI in males, and with height in both sexes. However, the increases in risk were modest.

## Figures and Tables

**Table 1:** Number of observed cases of small intestine cancer, person-years and overall incidence rates

	**Men**			**Women**		
**Variable**	**No. of cases**	**Person-years**	**Incidence rate^a^**	**No. of cases**	**Person-years**	**Incidence rate^a^**
*Time since measurement*						
0–4	42	4 704 799	1	36	5 104 845	1
5–9	55	4 186 615	1	62	4 630 922	1
10–14	84	3 539 359	2	78	4 042 558	2
15–19	77	2 974 592	3	97	3 554 864	3
20–24	107	2 514 913	4	92	3 134 983	3
25–29	101	2 100 174	5	108	2 716 707	4
⩾30	105	1 538 398	7	118	2 159 852	5
						
*Age at measurement*						
20–29	45	4 858 466	1	50	5 599 610	1
30–39	102	4 647 809	2	106	5 282 390	2
40–49	182	6 530 026	3	170	7 043 688	2
50–59	157	3 458 166	5	155	4 467 454	3
60–69	72	1 703 355	4	97	2 423 560	4
70–74	13	361 029	4	13	528 029	2
						
*Year of birth*						
<1900	17	468 357	4	19	686 032	3
1900–1909	67	1 775 881	4	94	2 555 664	4
1910–1919	157	3 514 584	4	161	4 554 801	4
1920–1929	177	4 995 227	4	156	5 576 162	3
1930–1939	91	4 317 744	2	97	4 836 647	2
1940–1949	48	4 646 655	1	52	5 114 247	1
⩾1950	14	1 840 403	1	12	2 021 176	1
						
*BMI (kg/m*^*2*^)						
<18.5	4	138 630	3	10	549 699	2
18.5–24.9	281	12 576 460	2	301	14 439 090	2
25.0–29.9	247	7 840 365	3	196	7 436 780	3
⩾30.0	39	1 003 396	4	84	2 919 162	3
						
*Height (cm)* b						
<150				9	367 010	2
<160/150–159	2	143 607	1	177	7 051 248	3
160–169	102	3 323 991	3	328	14 611 055	2
170–179/⩾170	307	11 763 593	3	77	3 315 417	2
⩾180	160	6 327 660	3			
						
Total	571	21 558 851	3	591	25 344 730	2

a Number of cases of small intestine cancer per 100 000 person-years.

b Split categories pertain to men and women, respectively.

**Table 2:** Relative risk (RR) of cancer of the small intestine with 95% confidence intervals (CI) obtained in Cox's regression analyses; age at measurement and birth cohort were included in the model in addition to either body mass index (BMI) or height

	**Cancer of the duodenum**		**Cancer of the small intestine**	
	**RR**	**95% CI**	**RR**	**95% CI**
*Men*	118 cases		571 cases	
BMI (kg/m^2^)				
<18.5	0.00	0.00-	1.53	0.57–4.11
18.5–24.9	1.00	Referent	1.00	Referent
25.0–29.9	1.18	0.80–1.72	1.22	1.03–1.45
⩾30.0	1.56	0.74–3.29	1.59	1.13–2.23
Test for trend^a^	*P*=0.1		*P*=0.02	
				
Height (cm)				
<160	1.03	0.14–7.50	0.45	0.11–1.83
160–169	0.99	0.60–1.61	0.99	0.79–1.25
170–179	1.00	Referent	1.00	Referent
⩾180	1.04	0.69–1.62	1.23	1.01–1.50
Test for trend^a^	*P*=0.7		*P*=0.01	
				
*Women*	112 cases		591 cases	
BMI (kg/m^2^)				
<18.5	0.78	0.11–5.65	1.20	0.64–2.25
18.5–24.9	1.00	Referent	1.00	Referent
25.0–29.9	1.10	0.71–1.70	0.94	0.78–1.13
⩾30.0	1.67	1.00–2.80	0.98	0.76–1.26
Test for trend^a^	*P*=0.2		*P*=0.2	
				
Height (cm)				
<150	0.00	0.00-	0.82	0.42–1.61
150–159	0.94	0.62–1.44	0.91	0.75–1.09
160–169	1.00	Referent	1.00	Referent
⩾170	1.33	0.74–2.39	1.35	1.05–1.74
Test for trend^a^	*P*=0.4		*P*=0.01	

a BMI and height, respectively, were included as continuous variables.
